# Proof-of-Concept Development and Preclinical Evaluation of a Microarray Patch Platform for Codelivery of Multiple Broadly Neutralizing Antibodies for HIV Prevention

**DOI:** 10.1021/acs.molpharmaceut.5c01650

**Published:** 2026-07-14

**Authors:** M. Melissa Peet, Vivek Agrahari, Parbeen Singh, Lam Luong Hoai Nguyen, Louise A. Ouattara, Eun Sung Yang, Carolina Herrera, Amarendra Pegu, Theodore C. Pierson, Thanh D. Nguyen, Gustavo F. Doncel

**Affiliations:** † CONRAD, 6042 Macon & Joan Brock Virginia Health Sciences, Old Dominion University, Norfolk, Virginia 23507, United States; ‡ Department of Biomedical Engineering, 7712 University of Connecticut, Storrs, Connecticut 06269, United States; § Vaccine Research Center, 35037 National Institute of Allergy and Infectious Diseases, National Institutes of Health, Bethesda, Maryland 20814, United States; ∥ VLP Therapeutics, Inc., Gaithersburg, Maryland 20878, United States; ⊥ Department of Mechanical Engineering, University of Connecticut, Storrs, Connecticut 06269, United States; # Institute of Materials Science, Polymer Program, University of Connecticut, Storrs, Connecticut 06269, United States

**Keywords:** broadly neutralizing antibodies, microarray patch, microneedles, transdermal delivery, HIV prevention in infants, antibody stabilization

## Abstract

Broadly neutralizing antibodies (bnAbs) are advancing as a preventative or treatment option for HIV infection, but currently must be administered parenterally, typically as a large injection or infusion, an invasive method of delivery, especially for the pediatric population. Here, we present proof-of-concept data on a minimally invasive microneedle (MN) patch platform codelivering three bnAbs, PGT121, VRC07-523LS, and PGDM1400LS, from separate MN patch units or pixels. Each 1 cm^2^ patch pixel, fabricated via solvent casting, contained ∼2 mg of one bnAb. In ex vivo porcine skin tests, the MNs effectively penetrated the skin. When tested in vitro in PBS buffer, MNs quickly dissolved, fully releasing bnAbs within 10 min. VRC07-523LS and PGT121 functionality was maintained during patch manufacturing, as confirmed by an in vitro HIV-1 neutralization assay, while PGDM1400LS lost some potency. In a rat model, coadministration of 1 cm^2^ patch units for each bnAb resulted in systemic exposure by the first collection time point of 3 h, reaching maximal concentrations by day 3 of up to 68 μg/mL. These preliminary findings suggest that the MN patch platform may serve as a promising alternative to injectable bnAb formulations for HIV prevention and treatment, particularly in neonates and infants, although further investigation is required to establish its clinical utility. Additionally, the solid-state MN formulation of bnAbs may eliminate the need for cold-chain storage or distribution, making the patch particularly suitable for use in resource-limited settings.

## Graphical abstract

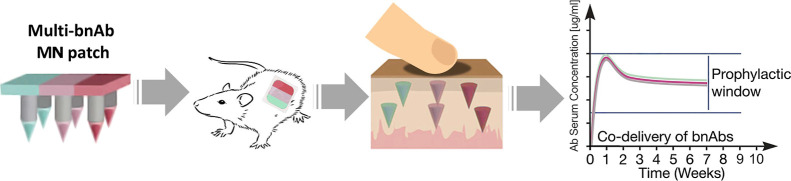

## 1 Introduction

In 2024, it was estimated that 40.8 million individuals were living with HIV worldwide, including 39.4 million adults and 1.4 million children.^1^ Each year, an estimated 1.3 million women and girls living with HIV become pregnant worldwide, and without appropriate interventions, the risk of mother-to-child transmission of HIV during pregnancy, labor, delivery, or breastfeeding is estimated to range between 15% and 45%.^1^ In pediatric populations, antiretroviral (ARV) therapy (ART) is highly effective; however, delivery of these drugs presents challenges due to poor palatability, complex regimens, frequent dosing, inadequate supply chain, and cold-chain storage requirements for liquid ARVs, resulting in poor access and adherence, and consequently, inadequate virus control, increased viral resistance, and reduced survival.^2^ These limitations underscore the need for more effective and longer-acting therapeutics and user-friendly formulations for the pediatric population.

In this population, the options available for HIV prevention are limited. Broadly neutralizing antibodies (bnAbs) are being investigated as additional or complementary tools for HIV prevention, demonstrating favorable safety, tolerability, and longer PK profiles in clinical trials of adults.^3–5^ They offer passive immunity for immediate protection and target conserved parts of HIV that are shared across many strains. For example, VRC01, a bnAb targeting the CD4 binding site of the HIV-1 envelope glycoprotein, demonstrates broad in vitro neutralization across all major circulating HIV-1 subtypes.^6^ bnAbs not only neutralize HIV-1 virions but may also mediate clearance of infected cells and augment both humoral and cellular immune responses.^7–9^ Furthermore, Fc-engineered versions, such as mutated bnAbs (LS-modified bnAbs), further extend antibody half-life and, when administered in combination, result in less frequent dosing and improved adherence.^10^

bnAb combinations targeting different epitopes provide better neutralization coverage.^11,12^ The combination of VRC07-523LS, PGT121, and PGDM1400 has been predicted to neutralize 99% of viruses and to have 7.3-fold greater efficacy than VRC01 alone with a PT_80_ > 200.^13,14^ A phase 1 trial (HVTN 130/HPTN 089) has explored this triple combinational therapy in adults and demonstrated its safety, tolerability, and lack of PK interactions or loss of neutralization.^13^ We have therefore selected these three bnAbs as prototypes for this proof-of-concept (POC) microneedle (MN) patch (also known as microarray patch) formulation development and preclinical testing, ultimately aiming to develop a new platform to prevent HIV acquisition in the pediatric population, especially breastfeeding neonates and infants.

Overall, the development of bnAbs diversifies and enhances existing preventive approaches, particularly for vulnerable populations. However, a limitation of bnAbs as an intervention remains related to their method of delivery. bnAbs are typically administered by parenteral injections because their large protein structure is not suitable for oral administration.^15^ The clinical use of injectable bnAbs in pediatric populations may be constrained by invasiveness and pain, cold-chain storage requirements, and the need for frequent dosing and administration by healthcare professionals. Therefore, developing alternative delivery platforms such as self-administrable, thermostable transdermal patches that provide immediate and/or controlled release of antibodies is critical to improving the accessibility of bnAb interventions.

MN patches have emerged as a promising platform for the systemic delivery of both small molecules and biologics.^16–18^ Evidence of dissolvable MN patches for vaccine delivery is advancing, eliminating the need for traditional hypodermic needles and cold-chain refrigeration. A clinical study completed in 2016, TIV-MNP 2015 (NCT02438423), demonstrated that an influenza vaccine could be delivered using the MN patch platform to adults safely and effectively generating immunity similar to the standard parenteral route.^19^ The minimally invasive nature of this platform and simplified administration makes it especially appealing for pediatric use. A recently published clinical trial in The Gambia (Pan African Clinical Trials Registry PACTR202008836432905) represents an important milestone in MN patch technology, demonstrating for the first time that a dissolving MN patch can safely and effectively deliver a measles and rubella vaccine to toddlers and infants while eliciting immune responses comparable to standard subcutaneous injection.^20^ These studies provide clinical POC for delivering biologics via dissolving MN patches, paving the way for future applications of this platform for other biological products such as bnAbs.

These easy-to-administer patches are designed to painlessly penetrate the outer skin barrier and deliver therapeutics into the dermis.^21^ Compared to injectables, MN patches eliminate the production of sharp biohazardous waste, reduce healthcare risks and burdens, and, due to their solid-state nature, may eliminate the need for cold-chain storage and transportation.^21,22^ Despite their potential advantages, MN patches remain largely unexplored for infant drug delivery, and, to our knowledge, only a limited number of studies have investigated the delivery of antibodies or bnAbs using MN patches.^23–27^ This gap is primarily due to the low loading capacity of antibodies in MNs and the difficulty of maintaining antibody structural stability and functionality during fabrication, storage, and application. Often, MNs have a modest drug loading under 1 mg per cm^2^ patch.^24^ Our previous works^24,28–30^ have shown that MN patches can successfully deliver high amounts of macromolecules, while preserving their bioactivity under ambient conditions.^31^ Yet, these reported MNs have not been shown to simultaneously deliver multiple clinically relevant HIV antibodies. The development of bnAb-loaded MN patches could therefore lead to a thermostable, cost-effective, minimally invasive, and user-friendly formulation for HIV prevention, particularly suited for the pediatric and neonatal population.

In this project, we developed MN patches for the intradermal delivery of three bnAbs (VRC07-523LS, PGT121, and PGDM1400LS) for potential use in neonates and breastfeeding infants for HIV prevention and tested their combination to demonstrate POC for the delivery of multiple bnAbs using this platform.^3,32^ MN patches, measuring 1 × 1 cm, were developed individually for each bnAb. These individual MN patches, called “pixels,” were designed to codeliver three different bnAbs. To enable simultaneous transdermal administration of more than one bnAb, multiple pixels can be arranged on a single MN patch backing layer, allowing for concurrent delivery. For example, a 2 × 2 cm patch can include four 1 × 1 cm pixels, each containing a different bnAb. To our knowledge, this is the first report demonstrating the simultaneous delivery of multiple bnAbs using an MN patch, employing an optimized solvent-casting method to improve bnAb loading efficiency (∼2 mg/1 cm^2^ size MN patch) while in general maintaining bnAb functionality during patch manufacturing. The MNs demonstrated sufficient mechanical strength to penetrate porcine skin (ex vivo testing) and quickly dissolved to provide a complete release of antibodies within 10 min in vitro. In a rat model, bnAb administration via MN patches provided systemic exposure by the first collection time point of 3 h, reaching maximal concentrations by day 3 of up to 68 μg/mL. This MN delivery platform offers a potential promising strategy for neonates and breastfeeding infants by overcoming key limitations of injectable formulations, including injection-associated pain, dependence on trained healthcare providers, and the generation of sharp biohazardous waste. While encouraging, these findings are preliminary, and further preclinical and clinical studies are needed to fully assess the platform’s safety, efficacy, and potential for translation to neonatal and infant populations.

## 2 Experimental Section

### 2.1 Materials

Poly­(lactic-*co*-glycolic acid) (PLGA), polyvinylpyrrolidone (PVP), rhodamine B, and ethanol were purchased from Sigma-Aldrich (St. Louis, MO). Sucrose and Pierce bicinchoninic acid (BCA) protein assay kit was purchased from Thermo Fisher Scientific (Waltham, MA). Polydimethylsiloxane (PDMS) molds were purchased from Dow Chemical (Midland, MI). PGDM1400LS and VRC07-523LS were provided by the Vaccine Research Center (VRC), the National Institute of Allergy and Infectious Diseases (NIAID, Bethesda, MD), and PGT121 was provided by the International AIDS Vaccine Initiative (IAVI, New York, NY). All other reagents were of analytical grade and used without further purification.

### 2.2 Fabrication and Characterization of bnAb-Loaded MN Patches

#### 2.2.1 Manufacturing of MN Patches

MN pixels containing PGDM1400LS, VRC07-523LS, or PGT121 were fabricated by solvent casting (Figure 1). Briefly, lyophilized bnAbs were reconstituted in water with sucrose (0.5 M) and PVP (10% solution, 40 kDa molecular weight) by vortexing for 3 min to form concentrated solutions containing 4 mg of each bnAb. These were cast onto PDMS molds, allowed to settle, and subsequently dried under vacuum. The casting and drying steps were repeated until the full bnAb load was incorporated into the molds, followed by overnight drying under ambient conditions. After drying, a PLGA (200 μm thick, 35–40 kDa molecular weight) backing layer was applied to the dried MNs through thermal lamination at 80–90 °C for 15 min, and the arrays were carefully demolded. Rhodamine B dye-loaded MN patches were also prepared for ex vivo testing in porcine skin using a similar method, except that a 10% (w/v) casting solution of dye was prepared in PVP through homogenization. Representative images of bnAb-loaded MN patches were acquired using an optical microscope (ME300TZC-2 L-8 M, AmScope, China) equipped with a digital camera (MU800, AmScope).

**Figure 1:**
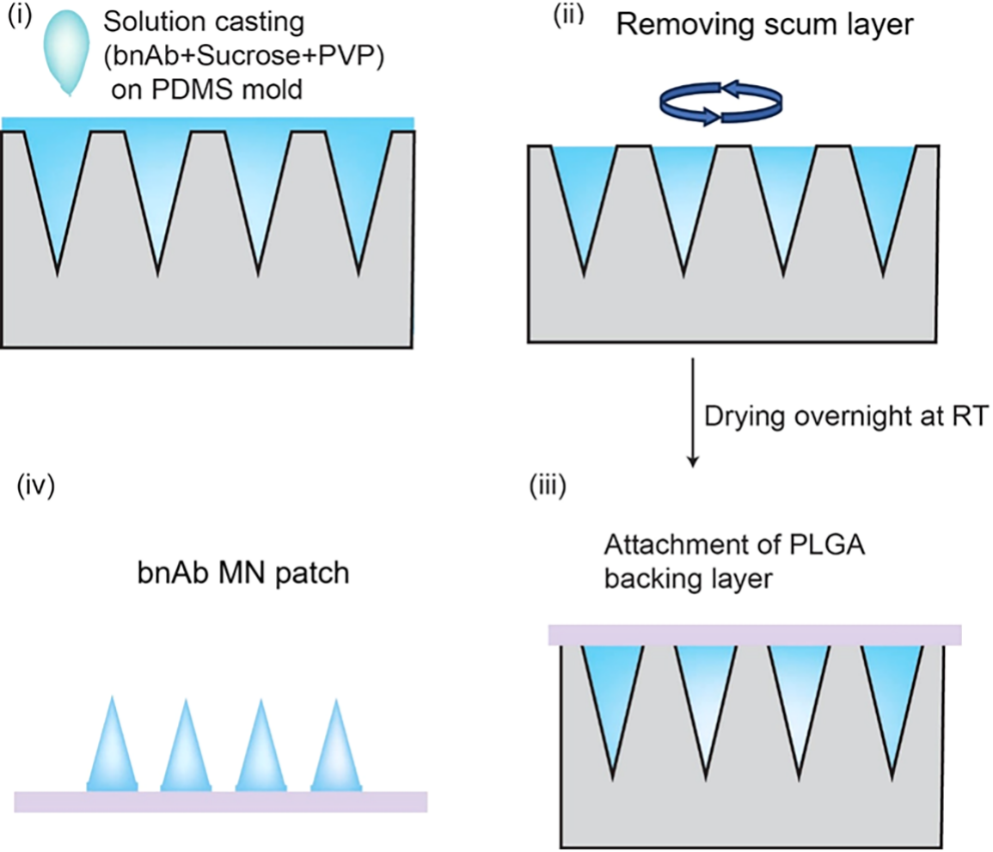
Schematic of MN patch fabrication using solvent casting method.

#### 2.2.2 bnAb Loading Efficiency in the MN Patch

To determine bnAb loading efficiency, MN patches (1 cm^2^) were dissolved in 1 mL water, casted onto the PDMS mold, and the total bnAb content was quantified using a Pierce BCA protein assay according to the manufacturer’s instructions (Thermo Fisher Scientific, Waltham, MA). The loading efficiency was expressed as the proportion of bnAb recovered from the patches relative to the total amount initially incorporated into the PDMS molds.

#### 2.2.3 In Vitro bnAb Release Study

Individual 1 cm^2^ size MN patches loaded with each bnAb were placed in 1 mL of phosphate-buffered saline (PBS) and incubated at 37 °C in a shaking water bath (50 rpm) to simulate physiological conditions. The supernatant was collected and analyzed using the BCA protein assay. The % cumulative release of each bnAb was subsequently determined by quantifying the recovered antibody concentration against a standard calibration curve (25–2000 μg/mL, *R* ^2^ > 0.99).

#### 2.2.4 Mechanical Testing of MNs

An EZ-LX mechanical tester (Shimadzu, Japan) equipped with two aluminum grips in its upper and lower clamps was employed to characterize the mechanical properties of the MNs.^28^ The patches (1 cm^2^) were affixed to the top of the lower aluminum grips. The upper clamp piston was gradually lowered to a position just above the tips of the patch, applying compressive force at a rate of 1 mm/min. The compression test was stopped when a compressive force of 50 N was reached, and the data were plotted as compression force per MN as a function of the displacement of the upper piston.

#### 2.2.5 Ex Vivo Insertion Testing of MNs on Porcine Skin

MN patches loaded with rhodamine B dye were manually applied to excised porcine skin using a commercially available spring-loaded applicator (Millipore Sigma, Burlington, MA). The applicator is designed to deliver a controlled and reproducible insertion force to the skin surface by incorporating a calibrated spring mechanism that ensures consistent impact velocity and insertion force across applications, thereby minimizing user-dependent variability. The same applicator was used for both ex vivo and in vivo studies. The patches were left in place for ∼10 min, allowing the MNs to fully dissolve under the skin. Following removal of the patch backing layer, the treated skin was washed with PBS and imaged with a digital camera (AmScope, China).

### 2.3 In Vitro Antiviral Activity before and after Patch Manufacturing

The functionality of the bnAbs preformulation (unformulated control) and postmanufacturing conditions (dried bnAbs and excipients exposed to heat, 80 °C for 20 min) was evaluated using a virus neutralization assay, performed as previously reported.^24^ Samples were diluted in Dulbecco’s minimal essential medium (DMEM) (Sigma-Aldrich, Inc., St. Louis, MO) containing 10% fetal bovine serum (FBS) and antibiotics (100 U of penicillin/mL, 100 μg of streptomycin/mL) to achieve concentrations in the μg/mL range. Neutralization assays were performed using a standardized challenge viral titer. TZM-bl cells were cultured in DMEM, seeded in 96-well flat-bottom plates (10^4^ cells per well), and 24 h later, exposed to serial dilutions of native (unformulated) and formulated bnAbs mixed with HIV-1_BaL_ (5 × 10^3^ TCID_50_) prior to being added to the cells.^24^ Neutralization potency was assessed after 48 h of incubation using the Bright-Glo luciferase assay system (Promega, Madison, WI). Briefly, cells were washed with PBS and lysed with 100 μL of Glo lysis buffer. Lysate (50 μL) was transferred to a 96-well black microtiter assay plate, and 50 μL of Bright-Glo assay reagent was added. The extent of luciferase expression was recorded in relative light units (RLU) by using a Synergy HT Multi-Detection microplate reader (BioTek Instruments, Inc., Burlington, VT). The neutralization percentage was normalized relative to RLU values obtained for cells infected with the virus in the absence of bnAb (0% neutralization) and cells cultured in the absence of virus and bnAb (100% neutralization). Two independent experiments were run at least in duplicates.

### 2.4 Pharmacokinetics (PK) Study of bnAb-Loaded MN Patches in Rats

All animal procedures were approved by the Institutional Animal Care and Use Committee (IACUC) of the University of Connecticut (Protocol No. A24-038) and conducted in accordance with institutional guidelines. For animal testing, MN patches were sterilized using ultraviolet (UV) irradiation within a Class II biosafety cabinet. The patches were placed in sterile Petri dishes with the MNs facing upward and exposed to UV light (254 nm) for 20 min each side, maintaining a distance of approximately 10 cm from the UV source to ensure even exposure. Male Sprague-Dawley rats (200–250 g) were housed under controlled conditions and acclimated for at least 7 days prior to the experiment. The dorsal skin was shaved and depilated with a commercial hair-removal cream to prepare the site for MN application. While under anesthesia using isoflurane (4% for induction and 2% for maintenance), sterilized MN patches were manually applied to the treated skin using firm thumb pressure for 5–10 min to ensure consistent MN insertion. Each rat (*n* = 3) received six patches (two 1 cm^2^ patches per bnAb) for a total dose of ∼4 mg/bnAb. Sterile saline was administered subcutaneously as a negative control to one animal for safety and PK. Serial blood collections were performed at time zero (before application) and at 3 h, 1 day, 3, 5, 7, 14, 21, and 28 days post-MN application. Serum was collected from whole blood by centrifugation at 3000 rpm for 30 min and stored at −20 °C until analysis. At the end of the study, rats were euthanized via carbon dioxide (CO_2_) inhalation following approved ethical protocols.

Serum bnAb levels were quantified using plates coated with anti-idiotypic antibodies specific for each bnAb (VRC07-523LS, PGDM1400LS, PGT121).^33^ Three mouse anti-idiotypic monoclonal antibodies were utilized in this study: clone 5C9 (IgG2a) directed against VRC07, clone 9G3C8F4 (IgG1, kappa) against PGDM1400, and clone 9E9-2 (IgG1) against PGT121. Briefly, Nunc MaxiSorp (Thermo Fisher Scientific) plates were coated overnight with 200 ng/well of idiotypic antibody in PBS, washed with PBS with Tween 20 (PBS-T) five times, and blocked with a blocking buffer of tris-buffered saline with Tween 20 (TBS-T) with 5% milk and 2% bovine serum albumin (BSA) for at least 1 h. 5-fold serial dilutions of serum ranging from 1:20 to 1:12,500 were made in blocking buffer. For each sample, three consecutive serial dilutions were plated in duplicate. An antibody-specific standard curve (starting at 200 ng/mL with eight 2-fold serial dilutions) and positive, negative, and spiked controls were included on each plate. Serum was incubated on the plate for 1 h at room temperature (RT), followed by a PBS-T wash. Bound antibodies were probed with a horseradish peroxidase-labeled donkey antihuman IgG (1:10,000 dilution; Jackson ImmunoResearch) for 30 min at RT. The plate was washed, and 100 μL of SureBlue TMB (SeraCare, Gaithersburg, MD) substrate was added. Once color was developed (typically 15 to 20 min), the stop solution (100 μL 1 N H_2_SO_4_) was added, and the optical density at 450 nm was measured. Softmax Pro V5.2revC (Molecular Devices) software was used to calculate Ab concentrations. The limit of detection (LOD) of the assay was 0.1 μg/mL.

### 2.5 Statistical Analysis

All experiments were performed in triplicate (*n* = 3), and data are presented as mean ± standard deviation (SD), unless otherwise described. GraphPad Prism 10.4.2 (La Jolla, CA) was used to perform descriptive statistical analysis. For the neutralization assays, experiments were run at least in duplicate. Inhibitory concentrations of unformulated and formulated bnAbs were compared via two-tailed unpaired Student’s *t*-test; a *p* value <0.05 was considered statistically significant (GraphPad Prism 10.4.2).

## 3 Results and Discussion

### 3.1 Manufacturing and bnAb Loading of MN Patches

The MN patches encapsulating bnAbs were fabricated using a solvent-casting technique.^24^ Solvent casting was selected owing to its simplicity, reproducibility, and compatibility with large-scale manufacturing. Unlike lithography-based methods, which are labor-intensive and cost-prohibitive for translation, solvent casting enables rapid production while maintaining high fidelity in MN geometry and uniform distribution of the therapeutic cargo.^30,34^ Furthermore, this method enables the fine-tuning of material composition and mechanical properties, ensuring adequate structural integrity of the MNs during storage, skin insertion, and in vitro and in vivo testing. The excipients in the MN formulations were selected based on our previous work with biologics, including antibodies.^24,28,30^ Sucrose was used as a stabilizing agent to preserve the conformational integrity and bioactivity of the bnAbs during reconstitution and drying. This protective effect may arise from replacing water-antibody hydrogen bonds with sugar-antibody hydrogen bonds, thereby minimizing aggregation or denaturation.^35,36^ PVP (40 kDa) was selected as the primary matrix polymer because of its excellent biocompatibility, water solubility, and ability to form mechanically robust dissolving MNs.^37,38^ In our previous and current studies, PVP-based MNs exhibited faster dissolution and appropriate mechanical strength for skin insertion.^28,39^ A backing layer based on PLGA (35–40 kDa) was incorporated to provide structural support and improve patch handling while retaining sufficient flexibility for effective skin insertion, consistent with findings from our previous study.^28^

Each 1 cm^2^ MN patch pixel consisted of a 15 × 15 array of MNs (700 μm height, 400 μm base diameter with 5 μm tip diameter, 225 MNs in total), with an average loading of approximately 2 mg of each bnAb (VRC07-523LS, PGT121, and PGDM1400LS; Figure 2a) from the starting dose of 4 mg used in the patch manufacturing. The mean % loading efficiency (±SD, *n* = 3) of VRC07-523LS, PGT121, and PGDM1400LS was 51.28% ± 4.19, 52.90% ± 2.17, and 49.93% ± 5.13, respectively. Light microscopy and scanning electron microscopy (SEM) images are presented in Figure 2b,c, respectively, and MN geometry and dimensions in Figure 2d.

**Figure 2:**
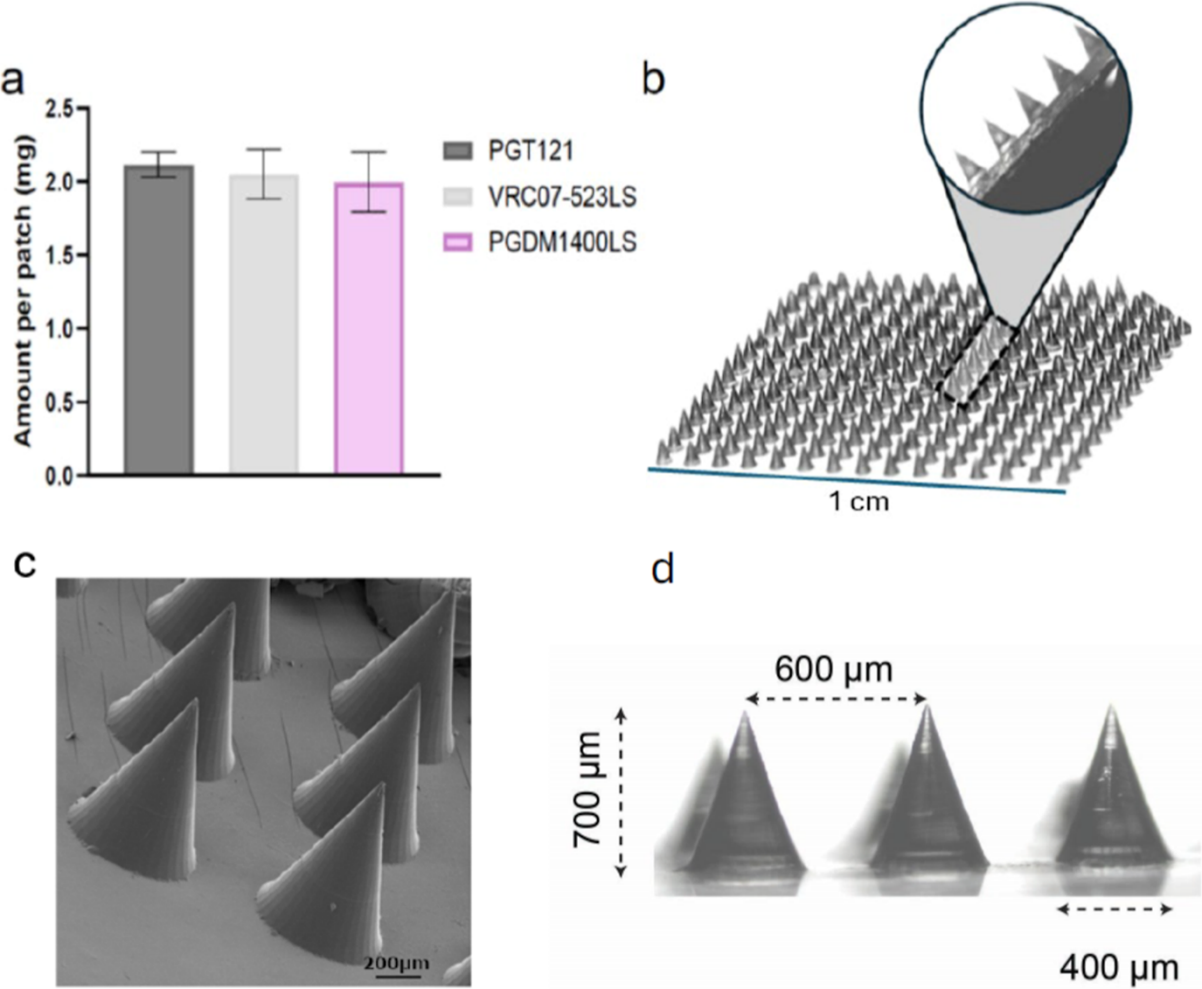
(a) Loading amounts of bnAbs in a 1 cm^2^ MN patch fabricated by the solvent casting method. Data are represented as means ± SD. (b) Microscopic image of a 1 cm^2^ patch containing a 15 × 15 array of MNs. (c) Representative SEM image of MNs from VRC07-523LS patch. (d) MN geometry and dimensions.

### 3.2 In Vitro bnAb Release from MN Patches

To demonstrate the immediate bnAb release from MN patches, separate 1 cm^2^ patches, each containing ∼2 mg of each bnAb, were tested in PBS buffer at 37 °C. As shown in Figure 3, MNs exhibited rapid release of bnAbs (∼100% within 10 min). The low molecular weight and amount of PVP used in MN fabrication facilitated rapid dissolution upon contact with the in vitro dissolution medium.

**Figure 3:**
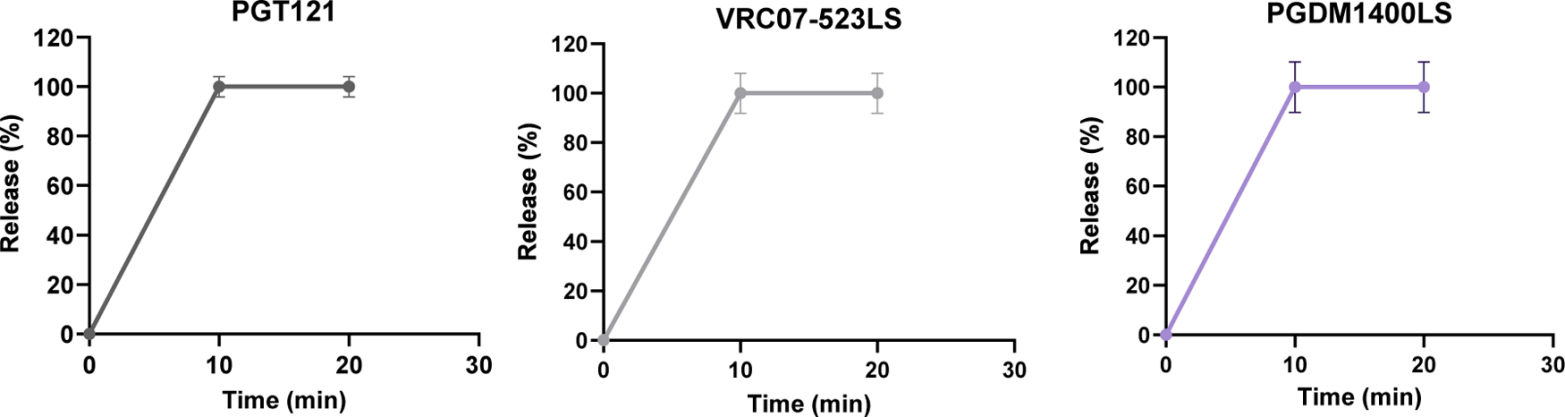
In vitro release of bnAbs from a 1 cm^2^ MN patch incubated in PBS buffer at 37 °C. Data are represented as means ± SD.

### 3.3 Mechanical Testing and Ex Vivo Penetration Testing of MN Patches on Porcine Skin

The mechanical strength and integrity of MN patches are a critical attribute for successful transdermal drug delivery, and MNs must withstand the compression force required to penetrate the skin without bending or fracturing. It was confirmed from the mechanical testing (Figure 4a) that all three bnAb-loaded MN patches can resist a total force of 50 N (∼0.22 N force per MN in case of 225 MNs per MN patch). From the literature and our previous experiments with other MN patches, a threshold insertion force of about 0.05–0.07 N has been established. Since our MNs tolerated a 50 N force, this confirms that the MNs were strong enough to penetrate human skin without damaging the array structure.^28,40^ The ex vivo penetration test of MNs loaded with rhodamine B dye on porcine skin (Figure 4b), selected for its structural similarity to human skin,^41^ further confirmed their mechanical stability for puncturing the skin to deliver antibody. These in vitro mechanical testing and ex vivo porcine skin penetration results represent screening-level evaluations rather than a comprehensive quantitative characterization of the MNs. Future studies will incorporate detailed quantitative assessments of these parameters to further validate MN performance.

**Figure 4:**
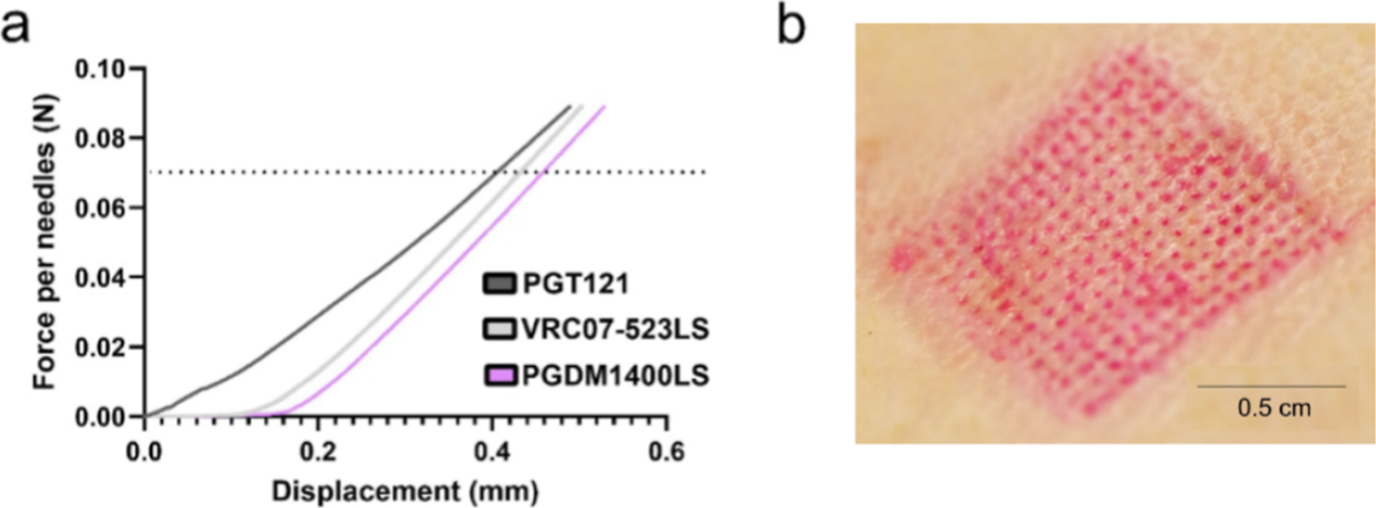
(a) Mechanical testing results of bnAb-loaded MN patches (1 cm^2^), and (b) ex vivo porcine skin penetration testing of MNs containing rhodamine B dye-loaded MNs (scale bar = 400 μm).

### 3.4 Antiviral Activity before and after Exposure to MN Patch Manufacturing Conditions

Neutralization potency of each bnAb was assessed pre- and postmanufacturing conditions to ensure that bnAb functionality was preserved during the manufacturing process. The results showed no significant loss of neutralization potency (Figure 5) for VRC07-523LS and PGT121 after their exposure to the formulation steps compared to unformulated bnAbs. This is in agreement with previous findings demonstrating conservation of another bnAb (b12) functionality after MN patch manufacturing.^24^ PGDM1400LS, however, showed some loss of neutralization potency after formulation, which was statistically significant (*p* = 0.03).

**Figure 5:**
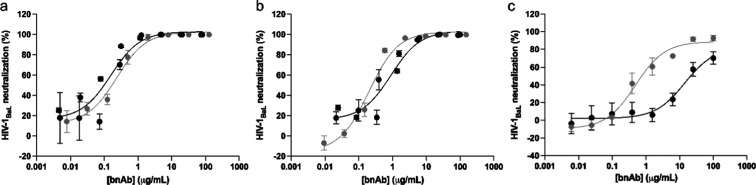
In vitro HIV-1_BaL_ neutralization activity of bnAbs before and after exposure to patch manufacturing conditions. TZM-bl cells were exposed to (a) VRC07-523LS, (b) PGT121, and (c) PGDM1400LS samples in the presence of HIV-1_BaL_. Neutralization potency was measured for the bnAbs unformulated (gray circles) and postmanufacturing conditions (black circles). Data are means ± SD from two independent experiments performed in at least duplicates.

### 3.5 PK Study of bnAb-Loaded MN Patches

To demonstrate the feasibility of the MN patch delivering three bnAbs simultaneously, concentrations of each bnAb were measured in serum collected from rats. All bnAbs were detectable in serum at the earliest time point of 3 h post patch application (Figure 6), reaching a maximum serum level of 68 μg/mL by day 3. Levels declined by day 14, likely due to a rat antidrug antibody (ADA) response against human antibodies, which accelerated their clearance.^42^ We observed a similar phenomenon in our previous study.^24^ As expected, non-LS PGT121 appears to drop to the LOD earlier than the two bnAbs, VRC07-523LS and PGDM1400LS, containing the LS mutation, which likely reflects faster natural clearance kinetics of PGT121.^43^

**Figure 6:**
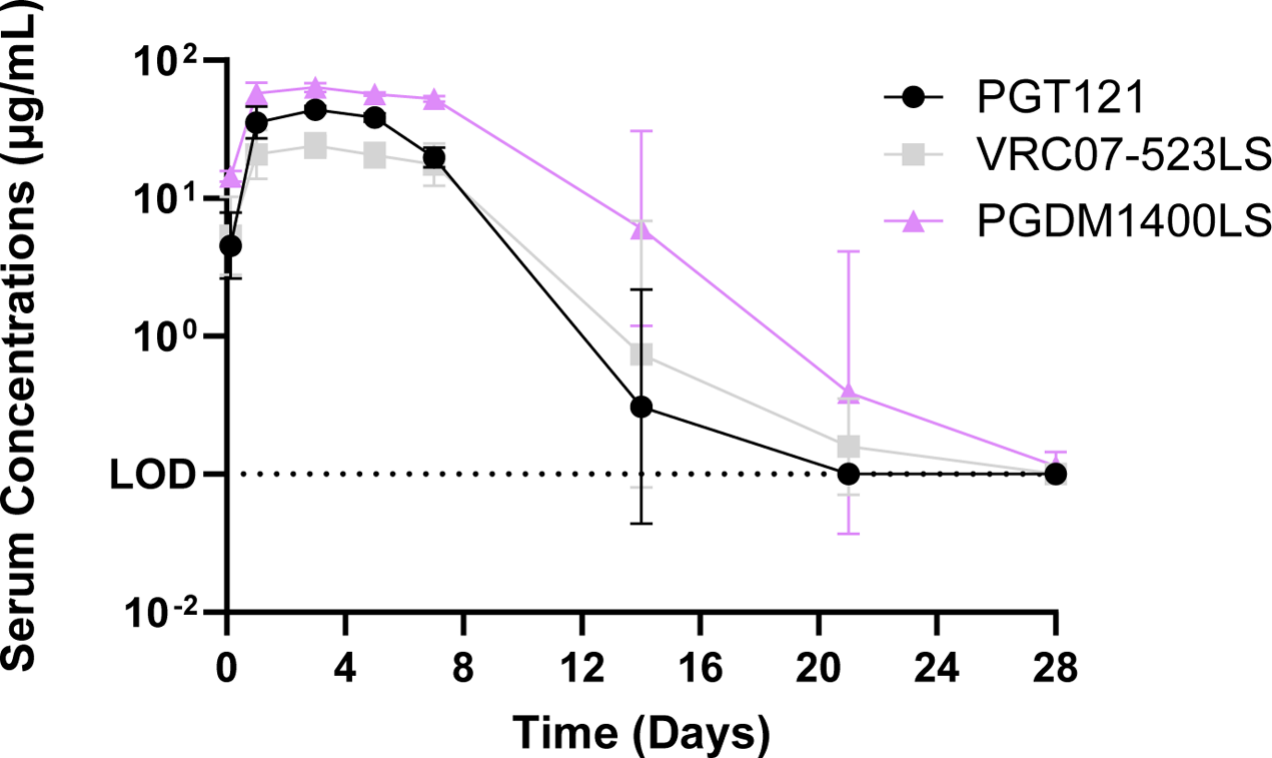
PK profile of simultaneous administration of three bnAbs in rats using separate 1 cm^2^ MN patch pixels. Data are presented in log scale (*y* axis) as means ± SD, *n* = 3 rats. The LOD is 0.1 μg/mL.

## 4 Conclusions and Future Directions

We present for the first time the development and 1 month preclinical evaluation of a MN patch platform simultaneously delivering three bnAbs for HIV prevention. Using a simple solvent-casting method, we fabricated noninvasive MN patches with a high bnAb loading capacity (∼2 mg in a 1 cm^2^ patch), achieving rapid in vitro release (∼100% within 10 min). The functionality of bnAbs was maintained throughout the fabrication process for VRC07-523LS and PGT121, as confirmed by comparative antiviral activity testing before and after exposure to patch manufacturing conditions. PGDM1400LS, however, lost some activity. It is possible that this bnAb is intrinsically less stable and that specific interactions with PVP may have diminished the stabilizing effect of sucrose during processing. We are currently investigating possible causes for the decreased neutralization potency of PGDM1400LS. In rats, coadministration of 1 cm^2^ patches of each bnAb resulted in detectable antibody concentrations in serum within 3 h of patch application. The MN patches are designed to be minimally invasive, self-administered, and fully dissolvable within minutes of skin application. Their high loading capacity allows for flexible dosing by adjusting the size or number of MNs per patch. Mechanical strength and ex vivo testing on porcine skin demonstrated that the MNs can reliably penetrate human skin without compromising their structural integrity. Additionally, the solid-state formulation of MNs may eliminate the need for cold-chain storage and distribution,^24,44,45^ and the fully dissolvable nature of the patches removes the risk of generating sharp biohazardous waste, unlike injectable options. Collectively, these features enhance user convenience, reduce healthcare burden, and offer a cost-effective approach to HIV prevention. Long-term stability studies under International Council for Harmonisation (ICH)-recommended accelerated and real-time storage conditions are necessary to confirm bnAbs’ integrity, potency, and final product shelf life. Beyond meeting these standard requirements, successfully translating this technology into the clinic requires navigating stringent regulatory authority (e.g., FDA) guidelines.

Developing combination biologics, like a bnAb MN patch, involves a complex FDA approval process because the product must meet the strict regulations of both a biologic and a medical device. This dual pathway requires extensive safety, manufacturing, and stability data, and adapting these products for pediatrics introduces even more hurdles. Researchers face ethical constraints, limited participant pools, and biological differences that prevent them from simply relying on adult data. Furthermore, regulatory mandates require dedicated pediatric study plans with age-appropriate dosing and formulations. Ultimately, navigating both the device-biologic requirements and these strict pediatric mandates significantly increases the time, cost, and evidence needed to prove the product is safe and effective.

To our knowledge, bnAb formulations under development have predominantly focused on parenteral routes of administration.^46^ Although alternative routes and delivery approaches, including intravaginal formulations (e.g., topical gels, films, and rings), intranasal delivery, and oral administration have also been investigated,^47–50^ these strategies may present challenges for effective dosing in pediatric populations. As bnAbs have become significantly more potent, the required dose volumes have decreased, enabling a critical shift from lengthy, invasive intravenous infusions to quick, simple subcutaneous injections.^5,51–53^ Efforts to enable sustained release and long-term immunoprophylaxis to reduce dosing frequency, have included subcutaneous delivery using polymer–nanoparticle hydrogels, liposomes, or hyaluronidase-enhanced administration of bnAbs.^5,54,55^ Standard subcutaneous administration, which limits injection volume to ≤2 mL, still requires multiple injections, posing a significant delivery challenge. With the development of more highly potent next-generation bnAbs and increased MN patch loading capacity, the potential to deliver these therapies via simple, noninvasive MN patches is within reach. Neonates and breastfeeding infants represent an ideal starting population to pioneer this technology, as their low body weights require a much smaller absolute dose. Compared to other antibody-based MN patches developed for other indications, the novelty of the MN platform discussed in this study relies on delivering functionally competent complex HIV bnAbs and achieving relatively high loading (∼2 mg per bnAb), which we are planning to further increase as discussed later in this section. High loading is especially advantageous for delivering potent HIV bnAbs, which are often administered in combinations of two or three, to neonates and infants requiring smaller-sized patches.

While we have successfully demonstrated POC development of bnAb-loaded MN patches, some limitations remain, warranting further investigation and optimization. A key area for improvement is to enhance loading efficiency (currently ∼50%) to minimize material loss, particularly during large-scale manufacturing. Improving loading efficiency would support cost-effective production, which is especially important given the high manufacturing costs and limited production yields associated with bnAbs. In addition, future efforts will focus on increasing bnAb loading (currently ∼2 mg) within a 1 cm^2^ patch to ensure adequate dosing while maintaining patch size and usability within neonates or infants.^56,57^ Although a 50% loading efficiency and a 2 mg loading capacity per 1 cm^2^ patch are generally considered competitive relative to previously reported dissolvable MNs,^25–27,58^ particularly for antibodies, which are challenging to incorporate due to their large molecular size, further improvements could reduce patch size and minimize material loss, thereby improving overall manufacturing efficiency and scalability. Potential optimization strategies to improve loading and dosing efficiency include localizing the bnAbs at the needle tips and employing solvent casting with highly concentrated bnAb solutions, which may increase the percentage loading by incorporating more antibody into the same mold volume.

In this POC study, the in vitro dissolution method was used to provide a preliminary assessment of MN matrix dissolution and antibody release. Future studies may incorporate Franz diffusion cell-based permeation and dissolution experiments to further validate these findings and enhance the translational relevance of the in vitro and ex vivo data. Also, in the current study, three bnAbs were administered using separate MN patches. Ultimately, all units are intended to be combined into a single multiunit patch to simplify dosing and potentially enhance user acceptability. Co-formulating all three bnAbs in a single MN matrix does not appear to offer significant advantages and would require additional formulation optimization and testing to ensure loading efficiency, release profiles, and compatibility among the three bnAbs. Our future research will focus on integrated MN designs capable of codelivering multiple bnAbs from different pixels in a single MN patch. The long-term storage stability of the MN patches under ICH-recommended conditions needs to be evaluated to ensure product integrity over time during storage and distribution. It is further essential to assess terminal sterilization methods that meet regulatory standards while maintaining bnAb structural integrity, bioactivity, and MN strength.

Preclinical development should progress to include evaluation of these bnAbs when delivered parenterally versus via MN patch, and further in neonatal and infant animal models to assess PK, safety, and immunogenicity, providing key translational data for clinical trials. This initial study did not include an injectable bnAb control, and consequently, absolute exposure, bioavailability, and other PK parameters cannot be quantitatively compared. These data serve as qualitative POC, demonstrating that MN patch administration successfully enables systemic delivery of bnAbs in a rodent model. The translational interpretation of these findings, particularly for the human pediatric population, requires caution due to the allometric and skin differences between the rat model and humans.^59^ Despite these limitations, our current findings demonstrate POC for the MN patch technology as a next-generation bnAb-based HIV treatment and prevention strategy, especially in neonates and infants. Furthermore, self-boosting MNs with time-dependent payload release may be developed to replace repeated patch applications.^24,28–30^ Our ongoing and future efforts will focus on addressing these challenges to advance the bnAb MN platform toward codelivery of multiple antibodies, scalable manufacturing, and clinical applications.
